# Posterior-Only Reduction of High-Grade Traumatic Thoracic Spondyloptosis

**DOI:** 10.7759/cureus.104311

**Published:** 2026-02-26

**Authors:** Paarth Patel, Thomas Tyler Patterson, Michael McGinity, Cristian Gragnaniello

**Affiliations:** 1 Department of Neurosurgery, University of Texas (UT) Health San Antonio, San Antonio, USA

**Keywords:** reduction, spine, spondyloptosis, technique, thoracic

## Abstract

Traumatic thoracic spondyloptosis is a rare, severe spinal injury that poses a major surgical challenge. Traditional approaches often involve complex mechanical maneuvers and increased patient risk. This case report describes a posterior-only reduction and stabilization technique using a distraction trauma system, monoaxial screws, and neuromonitoring, offering an alternative to more invasive strategies such as corpectomy or multi-rod constructs.

A patient with traumatic lateral T6-T7 spondyloptosis underwent single-stage posterior reduction using a spine distraction trauma system. The technique employed monoaxial pedicle screws and controlled application of multi-plane forces while avoiding early exposure of neural elements during reduction, reducing intra-operative risk. The spondyloptosis was reduced to a near-anatomic position, reversing approximately 12 mm of sagittal subluxation and 19 mm of coronal translation. Traumatic kyphosis improved from 24.6° pre-operatively to 7.8° post-operatively. Post-operative imaging confirmed maintained alignment, and neurological function showed continued improvement at follow-up.

This posterior-only technique enabled effective and controlled reduction of a high-grade fracture-dislocation without requiring anterior corpectomy or complex instrumentation. Compared to existing methods, such as Cobb elevator-assisted leverage, multi-rod constructs, or corpectomy, the described approach simplifies the surgical workflow. The use of monoaxial screws and a distraction trauma system may present a safe and reproducible strategy for managing severe thoracic spondyloptosis.

## Introduction

Spinal fractures are a common consequence of trauma, with recent estimates suggesting 8.6 million annual cases worldwide [1]. These injuries have been extensively classified. Denis's three-column system divides the spine into anterior, middle, and posterior columns, providing a framework to categorize fractures by morphology and mechanism, including compression, burst, and fracture-dislocation patterns [2,3]. These principles underpin widely adopted tools such as the Thoracolumbar Injury Classification System (TLICS) and the Thoracolumbar AOSpine Injury Score (TL AOSIS), which help guide surgical versus non-surgical management [4,5]. Although not developed specifically for trauma, Meyerding's classification of spondylolisthesis remains widely used, ranging from mild displacement (grade I) to spondyloptosis (grade V) [6]. Spondyloptosis is defined as a severe fracture-dislocation in which one vertebral body translates more than 100% beyond another, commonly associated with spinal cord injury and cerebrospinal fluid (CSF) leaks [7,8].

Transitional segments at the cervicothoracic and thoracolumbar junctions are more classically prone to injury due to their increased mobility, making them more vulnerable to fractures [9]. Some estimates suggest 75% of spine fractures occur between T10 and L2. The upper and mid-thoracic spine, however, is less susceptible to injury due to factors such as sturdy rib attachments, sternal bracing anteriorly, the sagittal orientation of facet joints, and robust ligamentous complexes [10]. Consequently, severe fracture-dislocations like spondyloptosis in the upper to mid-thoracic segments are typically seen only in high-energy trauma, such as high-speed motor vehicle collisions. Some even suggest that the level of force required to sustain upper to mid-thoracic spondyloptosis is incompatible with life [11].

The unique mechanics that make thoracic spine spondyloptosis rare also contribute to its challenging management. The ligaments, ribs, and facet orientations that normally add to thoracic spine rigidity make it difficult to achieve surgical reduction. The substantial forces required for reduction make these maneuvers technically demanding and potentially morbid. While a corpectomy may aid in reduction, it carries significant morbidity, particularly in the mid-thoracic spine. Previous descriptions have employed combined anteroposterior approaches, transpedicular anterior column reconstructions, quad-rod techniques, temporary rods, leveraged instruments, and manual extracorporeal pressure [10,12-15].

The objective of this report is to describe a posterior-only, single-stage technique for the controlled reduction of traumatic lateral thoracic spondyloptosis. This method emphasizes controlled distraction and gradual correction, representing a feasible alternative to more invasive approaches. To our knowledge, no prior reports have described a controlled correction of lateral thoracic spondyloptosis using this strategy.

## Case presentation

**Figure 1:**
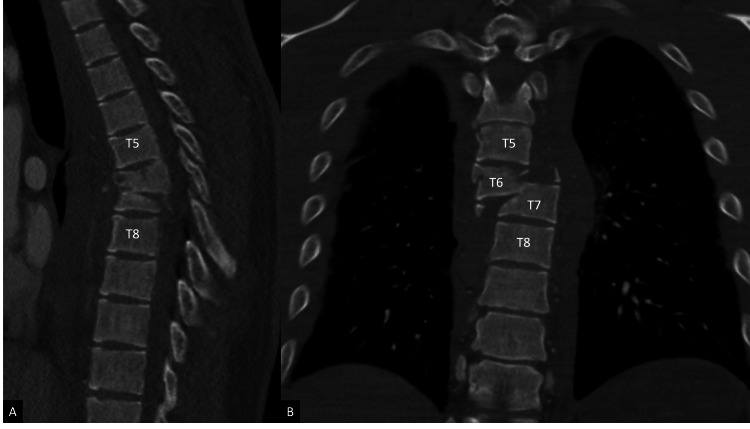
Pre-operative computed tomography scan of the thoracic spine. (A) Sagittal computed tomography scan demonstrating intact T5 and T8 vertebrae. (B) Coronal computed tomography scan demonstrating rightward lateral spondyloptosis of T6 on T7 with greater than 50% caudal translation of T6 relative to T7. T5, T6, T7, and T8 indicate thoracic vertebral levels.

**Figure 2:**
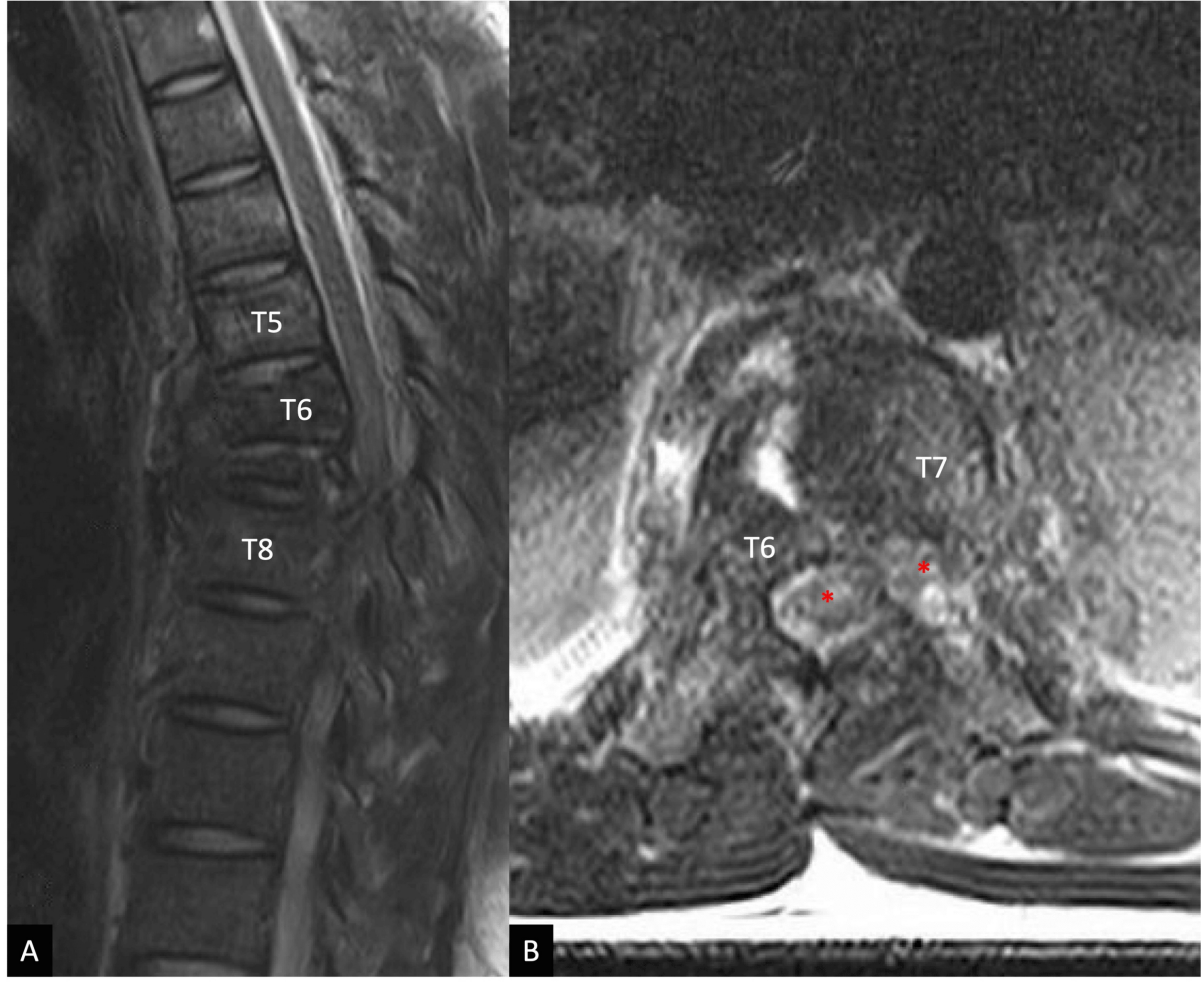
Pre-operative T2-weighted magnetic resonance imaging of the thoracic spine. (A) Sagittal magnetic resonance imaging demonstrating signal changes at the level of the traumatic injury. (B) Axial magnetic resonance imaging demonstrating a dual spinal canal with neural elements, consistent with stretch and/or transection injury to the spinal cord. Two red asterisks label each component of the dual spinal canal. T6 and T7 indicate thoracic vertebral levels.

A 27-year-old male patient with no medical history presented to our level I trauma center following a helmeted high-speed motorcycle collision. He arrived with bilateral lower extremity paralysis, absent rectal tone, and a T10 sensory level, consistent with an American Spinal Injury Association (ASIA) B spinal cord injury. Computed tomography (CT) demonstrated severe three-column thoracic fractures spanning T6-T8 with grade V lateral spondyloptosis of T6 on T7, characterized by rightward translation and caudal migration (Figure 1A, 1B). Magnetic resonance imaging (MRI) confirmed discal injury and ligamentous disruption across all three columns, near obliteration of the spinal canal, and a likely hematoma (Figure 2A, 2B). The patient also sustained extensive pulmonary, cardiovascular, and musculoskeletal polytrauma injuries.

After necessary resuscitation, the patient was taken to the operating room and placed prone for a posterior-only, single-phase operation. Intra-operative neuromonitoring was utilized throughout the operation. An open, midline approach was taken to the posterior spine. With the assistance of an intra-operative CT scanner, pedicle screws were placed using image-guided navigation bilaterally from T3 to T11. This was done without initial decompression to protect the spinal cord during reduction of the spondyloptosis. The left T6 and bilateral T7-T8 pedicles were not instrumented due to fractured pedicles that were not suitable for instrumentation. Monoaxial screws were used cranially and caudally to the spondylotic fracture at T5 and T8 levels bilaterally to facilitate distraction and realignment. Prior to any manipulation, appropriate screw placement was verified using intra-operative CT. 

**Figure 3:**
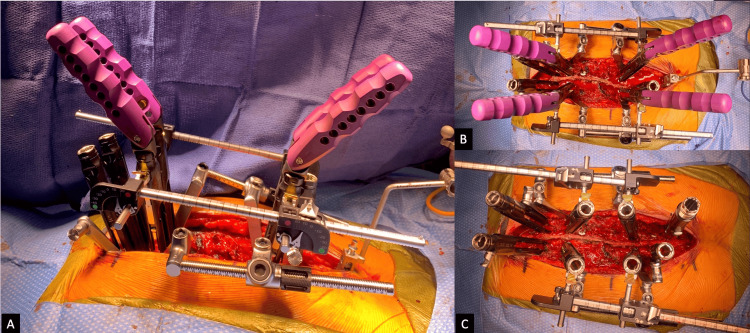
Intra-operative use of a distraction trauma system secured within pedicle screw towers cranial and caudal to the spondyloptosis, allowing controlled reduction through applied distraction forces. (A) Side view of pre-reduction setup. (B) Top view of pre-reduction setup. (C) Top view showing post-reduction alignment improvement.

Next, controlled distraction was performed using the Reline Trauma System (NuVasive Inc., San Diego, CA, USA) used according to manufacturer-intended indications (Figure 3A-3C). The system was anchored to the monoaxial pedicle screws at the T5 and T8 levels above and below the injury, respectively. The handles functioned as lever arms, generating opposing forces on the connected screws, and the setup allowed both independent distraction and angulation adjustments. Distraction was slowly performed under standard fluoroscopy until alignment between the two displaced spinal segments was achieved. Approximately 30 mm of distraction was necessary to allow enough space for the lateral relocation of the segments at T6 over T7. No distraction force measurements are available from this particular case. When satisfactory reduction was achieved, rods were placed bilaterally and held in place with set screws. A final intra-operative CT scan demonstrated that near-anatomic alignment had been achieved, with the successful reduction of the spondyloptosis. A posterior decompression was then performed from T6 to T8 to alleviate any pressure on the spinal cord. The case was concluded without complications. Estimated blood loss was approximately 1500 mL, and total operative duration was approximately nine hours.

**Figure 4:**
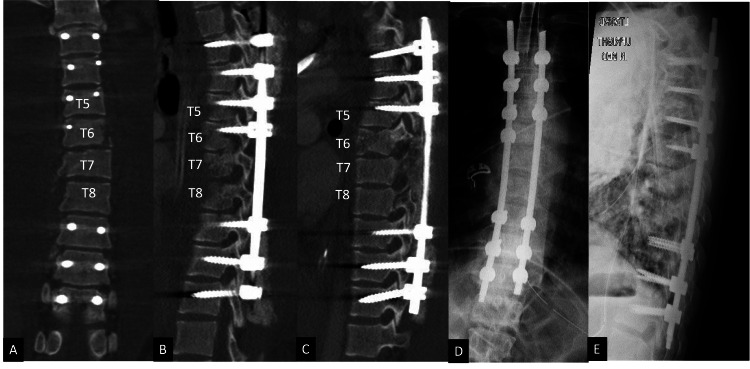
Immediate post-operative computed tomography and plain film radiographs of the thoracic spine demonstrating complete reduction of the T6-T7 spondyloptosis with the near-anatomic restoration of vertebral body alignment, bilateral facet joints, and the spinal canal. (A) Post-operative coronal computed tomography scan. (B) Post-operative right paramedian sagittal computed tomography scan. (C) Post-operative left paramedian sagittal computed tomography scan. (D) Post-operative upright anteroposterior plain film. (E) Post-operative sagittal plain film. T5, T6, T7, and T8 indicate thoracic vertebral levels.

**Figure 5:**
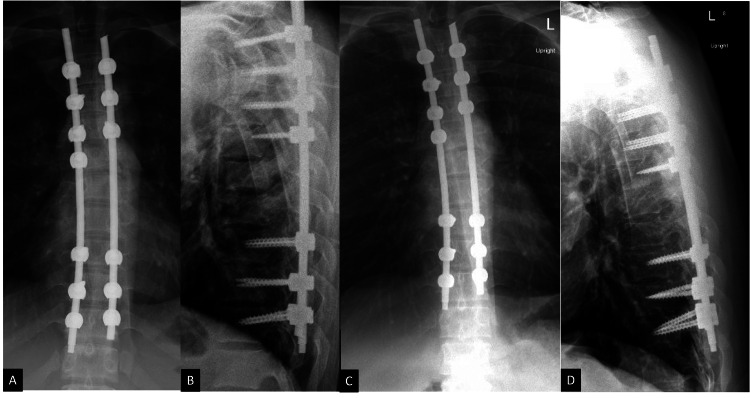
Post-operative plain films at the two-month and 15-month follow-up visits demonstrating stable alignment with no significant interval change. (A) Two-month post-operative upright anteroposterior plain film. (B) Two-month post-operative upright sagittal plain film. (C) Fifteen-month post-operative upright anteroposterior plain film. (D) Fifteen-month post-operative upright sagittal plain film.

Immediate post-operative CT scan and plain film radiographs confirmed the intra-operative findings of spondyloptosis reduction and near-anatomic alignment (Figure 4A-4E). The patient required extensive polytrauma management in the intensive care unit (ICU) and underwent several other non-neurosurgical procedures including intramedullary nailing of a right femoral fracture, external fixation for left tibial plateau and fibular injuries, and popliteal artery stenting. He required a prolonged 44-day hospitalization prior to transfer to inpatient rehabilitation. At the two-month post-operative clinic visit, he had improved clinically to ASIA C, with the ability to move his proximal right leg and sensory improvement. By six months post-operatively, he demonstrated further recovery with improved strength and voluntary movement throughout the right lower extremity. At 15 months, he had progressed to ASIA D, regained full bowel and bladder control, and improved to 3/5 strength in the right lower extremity, a marked improvement. Plain film radiographs at both the two-month and 15-month visits demonstrated stable alignment and no hardware complications (Figure 5A-5D).

## Discussion

Spondyloptosis of the thoracic spine presents unique management challenges. Traditionally, this severe three-column injury has been treated with combined anteroposterior reconstructions or instrumentation. A corpectomy with pedicle screw fixation has been reported through mostly combined approaches, though occasionally via a posterior-only (transpedicular) route [13,15-18]. While corpectomy can reduce the force required for reduction, it carries considerable morbidity, including increased operative time, blood loss, spinal column shortening, and potential injury to adjacent neurovascular structures. To avoid corpectomy, several alternative reduction techniques have been described. These include the use of a leveraged Cobb elevator, towel-clamp anchoring with distraction, and extracorporeal manual pressure [8,11,15]. These maneuvers may provide correction but also increase the risk of neural injury or CSF leak because they often involve levering against fragmented bone near the canal. In some cases, incomplete reduction and suboptimal alignment must be accepted [19].

More recently, posterior-only methods have emerged to optimize correction. One technique employed short horizontal temporary rods to apply sequential distracting forces across the injury [14]. While this allowed gradual realignment, its application is limited by the relatively narrow working distance of modern distracting tools, which restricts the number of levels that can be spanned and the amount of force that can be distributed. Another approach used two-headed screws for a quad-rod construct [12]. In this method, long-segment instrumentation spans above and below the spondyloptosis, while shorter temporary rods are positioned bilaterally without crossing the fracture. Reduction towers are then used to sequentially reduce the deformity, after which long permanent rods are secured. This technique distributes force across multiple pedicle screws, lowering the risk of screw pullout. However, it is mechanically complex, primarily facilitates distraction along a single mechanical axis, requires a wider exposure, and leaves a bulkier hardware profile that may cause post-operative issues.

In comparison, distraction systems such as the one used in this case offer distinct advantages. Monoaxial pedicle screws with dorsal guide towers allow the direct attachment of the system, and the independent tower connections enable multi-planar reduction. This facilitates both cranio-caudal distraction and lateral realignment simultaneously while avoiding corpectomy or anterior manipulation. Furthermore, permanent rods can be placed after reduction and realignment, eliminating the need to distract across temporary rods. In many spondyloptosis cases, temporary or quad-rod constructs do not align properly for set-screw placement. Multi-level force distribution also lowers the risk of hardware complications such as loosening or breakage. To our knowledge, there are no prior descriptions of posterior-only reduction of lateral thoracic spondyloptosis using a distraction system that permits independent tower movement to achieve multi-planar correction. Other distraction systems compatible with monoaxial pedicle screws and independently mobile tower interfaces may achieve similar correction.

Additionally, reduction can be achieved without exposing the thecal sac through laminectomy or facetectomy, which avoids the risk of early CSF leak and protects neural elements during alignment. Decompression can then be safely performed after fixation. Although some argue that decompression should not be delayed, timely reduction itself may relieve canal compromise. If preferred, decompression can still be performed first. Finally, this approach carries no significant opportunity cost. If the distraction system fails to achieve reduction, the surgeon can transition to an alternative technique to address the spondyloptosis. As a single-patient report, the reproducibility of this technique and long-term durability remain uncertain, and broader application should be interpreted cautiously.

## Conclusions

Thoracic spondyloptosis remains a challenging injury to manage surgically, often requiring complex maneuvers that increase operative morbidity. This case illustrates that controlled reduction may be achieved through a posterior-only, single-stage approach using distraction across monoaxial pedicle screws. By avoiding corpectomy, anterior exposure, or mechanically complex multi-rod constructs, this strategy allowed near-anatomic alignment and neurological improvement.

More broadly, the success of this method lies not in a single device but in the principle of distributing distraction forces evenly and allowing independent movement at each screw attachment. Techniques that incorporate these principles may provide surgeons with a potentially reproducible and less invasive option when addressing severe thoracic fracture-dislocations. As rare as spondyloptosis is, reports such as this aim to expand the spectrum of viable surgical strategies and highlight posterior-only distraction with multi-planar force distribution as a potential addition to the armamentarium for managing these devastating injuries. Further studies and attempts of this approach are necessary to portray the repeated viability of this strategy.
